# Effect of Thermal Stabilization on PAN-Derived Electrospun Carbon Nanofibers for CO_2_ Capture

**DOI:** 10.3390/polym13234197

**Published:** 2021-11-30

**Authors:** Elisa Maruccia, Stefania Ferrari, Mattia Bartoli, Lorenzo Lucherini, Giuseppina Meligrana, Candido F. Pirri, Guido Saracco, Claudio Gerbaldi

**Affiliations:** 1 GAME Lab, Department of Applied Science and Technology, Politecnico di Torino, Corso Duca degli Abruzzi 24, 10129 Torino, Italy; elisa.maruccia@polito.it (E.M.); giuseppina.meligrana@polito.it (G.M.); 2 Center for Sustainable Future Technologies (CSFT), Istituto Italiano di Tecnologia (IIT), Via Livorno 60, 10144 Torino, Italy; mattia.bartoli@iit.it (M.B.); fabrizio.pirri@iit.it (C.F.P.); 3 National Reference Center for Electrochemical Energy Storage (GISEL)-INSTM, Via G. Giusti 9, 50121 Firenze, Italy; 4 Department of Pharmacy, Università di Chieti Pescara “G. d’Annunzio”, Via dei Vestini 31, 66100 Chieti, Italy; 5 Department of Applied Science and Technology, Politecnico di Torino, Corso Duca degli Abruzzi 24, 10129 Torino, Italy; lorenzo.lucherini@epfl.ch (L.L.); guido.saracco@polito.it (G.S.)

**Keywords:** poly(acrylonitrile) (PAN), electrospinning, carbon fiber, CO_2_ adsorption, renewable feedstock

## Abstract

Carbon capture is amongst the key emerging technologies for the mitigation of greenhouse gases (GHG) pollution. Several materials as adsorbents for CO_2_ and other gases are being developed, which often involve using complex and expensive fabrication techniques. In this work, we suggest a sound, easy and cheap route for the production of nitrogen-doped carbon materials for CO_2_ capture by pyrolysis of electrospun poly(acrylonitrile) (PAN) fibers. PAN fibers are generally processed following specific heat treatments involving up to three steps (to get complete graphitization), one of these being stabilization, during which PAN fibers are oxidized and stretched in the 200–300 °C temperature range. The effect of stabilization temperature on the chemical structure of the carbon nanofibers is investigated herein to ascertain the possible implication of incomplete conversion/condensation of nitrile groups to form pyridine moieties on the CO_2_ adsorption capacity. The materials were tested in the pure CO_2_ atmosphere at 20 °C achieving 18.3% of maximum weight increase (equivalent to an uptake of 4.16 mmol g^−1^), proving the effectiveness of a high stabilization temperature as route for the improvement of CO_2_ uptake.

## 1. Introduction

The majority of anthropogenic greenhouse gas (GHG) emissions are constituted by carbon dioxide (CO_2_) [1], that accounts for 65% of all gases emitted and has reached values higher than 400 ppm in the atmosphere since the beginning of the industrial revolution when it was just 278 ppm [2]. The observed increase of the global average temperatures since the mid-20th-century is very likely due to the continuous rise of the GHG emissions globally produced [2,3]. Moving away from the burning of fossil fuels for energy production toward renewable resources is undoubtedly the main path to follow in the long term to slow down and hopefully stop/invert climate change [4,5]. To mitigate the current emission level, other solutions are being considered, alongside clean energy production in the short term, such as CO_2_ sequestration [2,6]. In recent years, carbon capture and storage (CCS) technologies have undergone massive development and at present are the subject of many investigations worldwide directed at finding innovative and more effective approaches that demand low energy, along with easy and low-cost manufacturing [7]. Chemical absorption by aqueous amine-based solvents is a commercially available technology for post-combustion CO_2_ capture, but requires a vast amount of equipment and has a high cost due to the high parasitic energy consumption [8,9]. Alternative, more economic adsorption methods are considered very promising, such as those that make use of porous materials including zeolites, MOFs, silica, porous polymers and carbons. Activated carbons with a high surface area, porosity and good chemical stability are industrially available and have demonstrated good efficiency as adsorbents for H_2_, CH_4_ and CO_2_. However, carbon nanopowders need to be transformed into functional components such as membranes or films to be useful in practical applications; in addition, additives such as binders, and fabrication methods like hot pressing have to be used, which might affect their textural features. Among the various porous carbon nanomaterials, carbon nanofibers (CNFs) have been recently studied as effective CO_2_ adsorbent thanks to their morphology and intrinsic porosity [10]. Carbon cloth and mats can be easier to handle compared to nanopowders and offer more opportunities for designing conformable coatings or liners inside containers used in industrial plants [11], even if their cost is significantly higher than that of carbon felt [12].

Electrospinning has received growing attention in the last ten years thanks to its versatility in terms of processable materials [13]. Both academia and industry have found electrospinning to be very attractive for its simplicity and relatively high production rate [14]. Nanofiber mats can be obtained from a wide range of polymers, both natural and synthetic, as well as from ceramic materials [15]. Together with an increased surface area typical of nanoscale objects, spun mats of disordered fibers exhibit a porous and interconnected structure inherent to non-woven textures [16]. Moreover, material characteristics are combined with unique features emerging from the single fiber morphology and, on a larger scale, from the layered complex of spun fibers [17,18,19]. Electrospun mats are thus of enormous interest for applications where highly porous [20], interconnected and, if necessary, functionalized [21] structures are highly desirable, such as in energy storage [22], environmental [23], catalysis [24], and biomedical fields [25].

Air and water filtration are two major applications of non-woven mat thanks to their high porosity and high surface area which enable a higher number of interaction sites [26]. They show effective removal of micrometer size air pollutants particles via physical trapping and adsorption on fibers surface and less pressure drop across the membrane [27,28,29].

PAN-derived carbon fibers have been recently under investigation for gas adsorption [30,31,32], particularly because their properties are prone to being easily and finely tuned by means of thermal treatments carried out in different atmosphere (N_2_, Ar) and at different temperatures [33,34]. In addition to functional groups, the pore structure and the micropore surface area are far more significant than the BET area for increasing the gas selectivity of carbon nanofibers. The optimal processing parameters to enhance the adsorption ability of the fibers mats still need to be properly identified and have been a current matter of discussion [33,34,35,36]. The effect of carbonization temperature on the fibers adsorption properties has been recently explored [34,35,36], but further helpful information in determining how to design an optimal adsorbent for gases could be extracted by also investigating the impact of the stabilization step which is the initial thermal treatment in oxidative atmosphere. Herein, CNFs from electrospun polyacrylonitrile (PAN) are proposed for CO_2_ capture. Contrarily to the traditional CNFs production process [37], the proposed approach did not involve a final graphitization step, but it explored the differences induced during the stabilization. Accordingly, the carbon mats were produced by a simple, reproducible, and inexpensive procedure which did not require physical or chemical activation of the fibers to achieve good CO_2_ uptake values. Furthermore, some preliminary considerations on the effect of the stabilization step were also reported. Then, a tentative was made to correlate the thermal history of the stabilization step with the adsorption performance.

## 2. Materials and Methods

**Figure 1:**
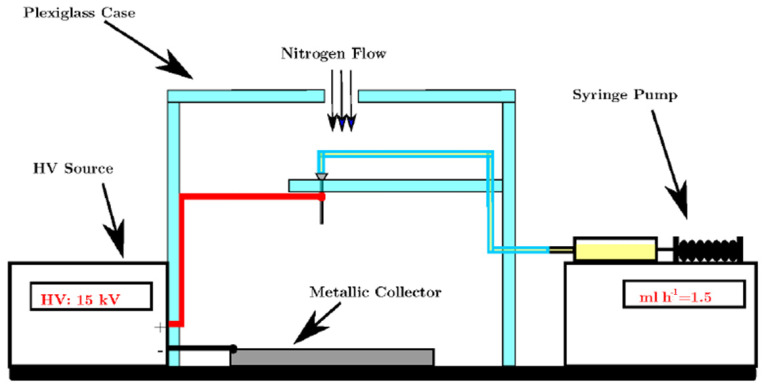
Schematic representation of electrospinning apparatus.

The electrospinning set up used in this work is shown schematically in Figure 1. The basic principle of this method is to induce static electrical charges on the molecules of the precursor solution such that when the charge repulsion is higher than the surface tension, the drop tip is broken and a continuous jet of polymer and solution is drawn towards the current collector by the electric field.

A plastic box surrounded the needle tip and the metallic target. Inside the box a thermometer and humidity sensor were used to monitor the environmental conditions. The humidity of the box was set to 40% by using nitrogen flow. A high voltage (HV) supply and a syringe pump were placed outside the box. A high-resolution camera was used to check the formation of the Taylor cone at the needle tip. Before starting the electrospinning process, a precursor solution was prepared by dissolving polyacrylonitrile (PAN) in dimethylacetamide (DMAc). The resulting solution was 7 wt.% PAN. A syringe connected to a metallic blunt-end needle was then loaded with the precursor solution and placed in a syringe pump for flux control. The needle pointed towards a flat metallic target and the high voltage source was connected between the two. The HV source was turned on up to a voltage of 15 kV and the syringe pump was operated at a constant flux of 1.50 mL h^−1^. The distance between the needle and collector (grounded, covered with Al foil) was 15 cm.

The stabilization of the structure of the fibers was carried out in the air, in a Thermo Scientific Heraeus oven with three slow ramps of temperature, first at 100 °C for 30 min, then 200 °C for 30 min and finally at 230 °C for 1 h or 260 °C for 2 h, thus obtaining the CNF_230 and CNF_260 samples. After stabilization, the samples were carbonized in a tubular furnace in Argon at 900 °C for 2 h, with a heating rate of 5 °C min^−1^. Some control samples were also considered (see Supplementary Information for more details).

Scanning Electron Microscopy (SEM) images were collected by using a Zeiss Gemini 300 SEM (Oberkochen, Germany) on samples coated with 5-nm-thick sputtered carbon.

The FTIR spectra were acquired by a Nicolet 6700 spectrometer in ATR mode (Thermo Fisher Scientific, Madison, WI, USA). Scans were acquired from 600 to 4000 cm^−1^. Baseline removal and peaks match were performed with Nicolet software. Raman spectra of CNF mat before and after carbonization were acquired with a Renishaw inVia microscope in the 250–4000 cm^−1^ range with an excitation wavelength of 532 nm.

X-ray Photoelectron spectroscopy (XPS) characterization was carried out with a PHI 5000 Versaprobe spectrometer (Physical Electronics, Chanhassen, MN, USA) using monochromatic Al Kα source (hν = 1486.6 eV). Survey spectra were collected with a pass energy of 160 eV over a binding energy (BE) range of 1200–0 eV. High-resolution spectra were obtained using a 20-eV pass energy (resolution approximately 0.4 eV). The spectrometer was calibrated using the C1s peak of graphite. Peak fitting was performed by using Origin 9 pro software (OriginLab Corp., Northampton, MA, USA), using mixed Gaussian (Voigt) line shapes and Shirley backgrounds.

CO_2_ adsorption isotherms were measured at 20 °C by using a TriStar II analyzer (Micromeritics Instrument Corporation, Norcross, GA, USA) after outgassing the samples at 150 °C under vacuum. Thermogravimetric analysis (TGA) performed on a NETZSCH TG 209 F1 Libra was used to assess the selectivity for CO_2_ adsorption by the electrospun mats in a simulated post-combustion flue gas mixture (20/80 *v*/*v*, CO_2_/N_2_) [38]. The CO_2_/N_2_ adsorption step (8 mL min^−1^ of CO_2_ in a total flow of 40 mL min^−1^) was carried out at 30 °C and ambient laboratory pressure (~1 bar). The samples were activated before each test under vacuum at 120 °C for 30 min in order to remove previously adsorbed gases or water.

## 3. Results and Discussion

### 3.1. Stabilization and Carbonization

**Figure 2:**
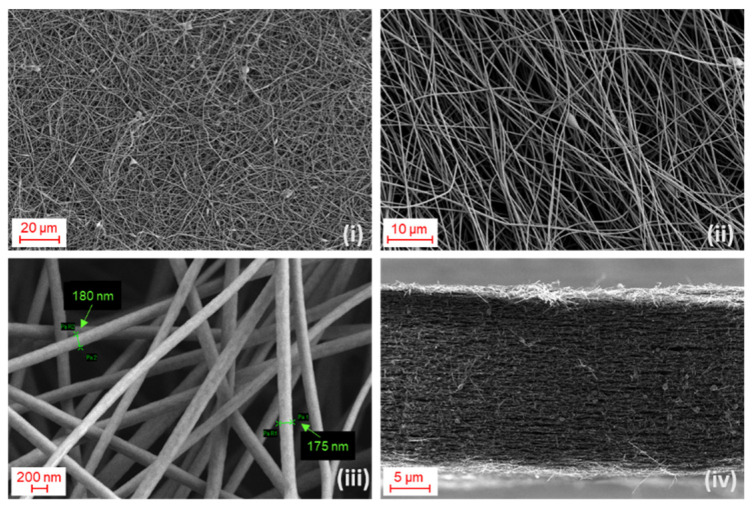
SEM images of (**i**,**ii**) as-spun carbon nanofibers at different magnifications; (**iii**) carbonized fibers with analysis of diameter; (**iv**) cross section of the mat.

Carbon fibers are well known as suitable materials to reinforce composites, and to this end, commercial nanofibers are very often produced by thermal treatment of PAN. PAN is a robust, high melting polymer that, when used as a precursor, gives carbon fibers with a higher performance, strength and stiffness compared to pitch, rayon etc. [32]. Indeed, the electrospun fibers showed exceptional mechanical stability that allowed easy handling. The morphological characteristics of the mats were evaluated by SEM (Figure 2).

The samples appeared as a non-woven mat of fibers with an estimated average diameter of about 320 nm. After the spinning, the second step was the stabilization of the fibers. The resulting chain structure guarantees an improved stability during carbonization and the final CNFs were more flexible. The carbonization process took place as soon as the stabilization ended to avoid moisture absorption. The set temperature was 900 °C and the carbonization took about 2 h to be completed. The morphology of the fibers (see Figure 2iii) was not affected after stabilization and carbonization, and the diameter was not significantly altered since an average value of 341 (47) nm was determined. The average diameter was reduced to 173 (14) nm after the carbonization step which is 45% of the pre-carbonization value. Weight loss of about 50% was also observed after carbonization. Both results were attributed to nitrogen and hydrogen loss during the high temperature treatment. The SEM image of the cross section of the carbonized mat is shown in Figure 2iv. The CNFs mat was characterized by multiple stacking of different sheets in which the fibers are randomly oriented. Cavities due to lack of contact among the fiber sheets gave rise to interconnected pores in the micron range distributed along the section.

**Figure 3:**
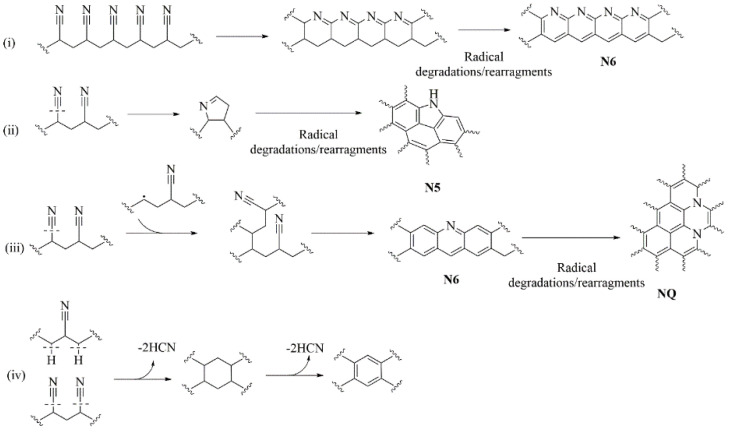
Proposed mechanism for (**i**) N6, (**ii**) N5, (**iii**) NQ and (**iv**) aromatic rings formation occurring during stabilization and carbonization of PAN fibers.

The proposed complex mechanism of stabilization and carbonization of PAN fibers is briefly represented in Figure 3.

**Figure 4:**
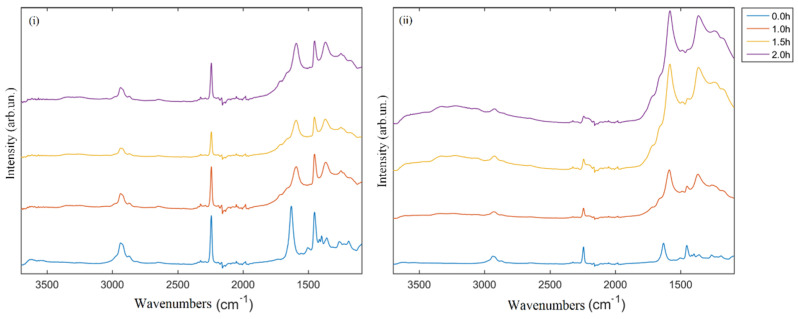
FT-IR (ATR mode) of electrospun CNFs precursor (0.0 h) then stabilized in air at (**i**) 230 and (**ii**) 260 °C for times ranging from 1 up to 2 h.

The thermo oxidative stabilization step in air is usually carried out below 300 °C as the first step which enables the formation of a ladder structure that does not collapse during the subsequent thermal treatments. Therefore, this stage controls and drives the final mechanical properties of the CNFs, and chemical properties, such as the elemental content of the carbon fibers, might also be influenced by this thermal treatment. After stabilization in air, the PAN structure could undergo several modifications due to the oxidation and rearrangement of nitrile groups with the production of condensed nitrogen-doped rings (Figure 3i) that could further evolve to N6 moieties [32,35]. Furthermore, homolytic cleavage of nitrile groups could also lead to the formation of N5 (Figure 3ii) and NQ (Figure 3iii) structures after dehydrogenation reactions. Interestingly, Grassie et al. [34] reported the formation mechanism of carbon rings through the coupling of homolytic nitrile cleavage and heterolytic hydrogen cleavage (Figure 3iv). The occurrence of these mechanisms was supported by the FT-IR spectra, which are shown in Figure 4.

**Figure 5:**
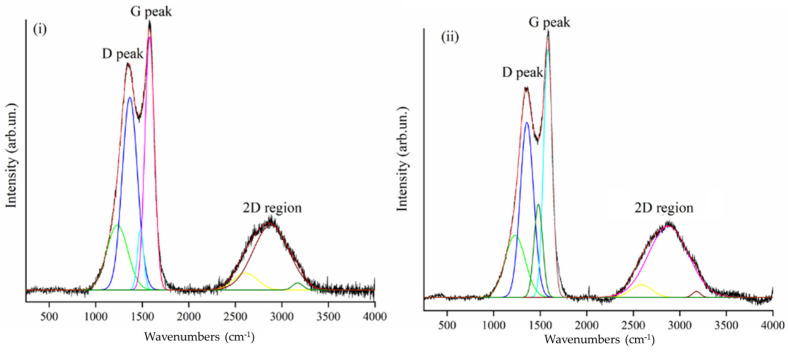
Raman spectra in the range from 500 cm^−1^ up to 4000 cm^−1^ of (**i**) CNF_230 and (**ii**) CNF_260.

CNFs stabilized at 230 °C display a reduction of peak of ν_CN_ centred at around 2240 cm^−1^ and a drastic lowering of ν_C=O_ peaked at around 1740 cm^−1^ that was likely due to the presence of carboxylic additives [39] in the commercial PAN. The ν_CH2_ signal at 1452 cm^−1^ is not significantly decreased after 1 h (sample CNF_230) and 2 h of treatment at 230 °C indicating that dehydrogenation was barely initiated. At 260 °C, ν_CN_ peak intensity is further reduced, suggesting an advanced nitrile groups rearrangement [39] with rising of ν_C=C_ and ν_C=N_. Stabilization time seemed to poorly affect PAN when a temperature of up to 230 °C is used during the stabilization process, while it became more relevant at 260 °C. After 2 h at 260 °C, ν_CN_ and ν_CH2_ peaks completely disappeared and the neat and nitrogen containing aromatic moieties are massively detected. After the stabilization, the pyrolytic treatment induced the conversion of PAN fibers to CNFs with the disappearing of residual functionalities and the ordering of carbonaceous structure, as clearly emerges from Raman spectra shown in Figure 5.

Both Raman spectra of CNF_230 and CNF_260 show sharp D and G peaks and a poorly structured 2D region supporting the formation of sp^2^ condensed disorganized structures. Nevertheless, the I_D_/I_G_ ratio of CNF_230 is considerably higher than CNF_260 reaching 1.34 and 1.04, respectively. This is in good agreement with the evolution of the PAN fibers shown in Figure 5, supporting the effectiveness of the stabilization process at 260 °C.

**Figure 6:**
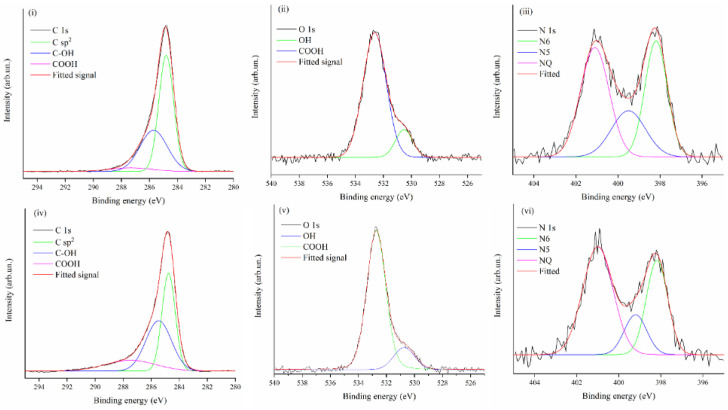
XPS spectra of CNF_230 (C 1s (**i**), O 1s (**ii**), and N 1s (**iii**)) and CNF_260 (C 1s (**iv**), O 1s (**v**), and N 1s (**vi**)).

The XPS spectra of CNF_230 and CNF_260 provide more detailed information about the residual groups retained by CNFs after pyrolysis, as shown in Figure 6 and summarized in Table 1.

The high resolution C 1s spectra of both CNF_230 and CNF_260 (Figure 6i,iv) show the typical sp^2^ carbon asymmetric peak centered at 284.8 eV together with a second component that according to some authors [40,41] can be attributed to C-X type bonds, like C-O and C-N bonds. The relative concentration of nitrogen on the surface was found to be 7.1 and 6.6 at.% in CNF_230 and CNF_260, respectively (XPS survey spectra in Figure S1 in the Supporting information) and the distribution of nitrogen species as extracted by XPS signal deconvolution is reported in Figure 6iii,vi. It was shown that nitrogen containing groups can have a great impact on the adsorption properties; an experimental and computational analysis revealed that the specific surface area is not as important as the N doping level on the CO_2_ uptake with N5 and N6 types showing great affinity for CO_2_ and large adsorption energies [42]. Those observations led to discussions around which type of group is the most relevant between pyrrolic and pyridinic for CO_2_ adsorption [32,35,43]. Therefore, XPS was useful to determine the relative amount of those N groups. CNF_230 showed percentages of N6 (pyridinic, 398.2 eV), N5 (pyrrolic, 399.5 eV) and NQ (quaternary, 401.1 eV) of up to 36, 22 and 43%, respectively. For the CNF_260 sample, a lower amount of N6 and N5 (32% and 14%, respectively) and a greater amount of NQ up to 53% were determined. Therefore, in these samples, both pyridinic and quaternary nitrogen groups are predominant, while the N5 groups are less representative, especially in CNF_260.

Interestingly, the nitrogen percentage of CNF_230 was higher than in CNF_260 (Figure S1). An opposite trend was observed for carbon, with percentages of 85.6 at.% for CNF_230 and 83.9 at.% for CNF_260. In agreement with the mechanism reported in Figure 3, we hypothesized that the higher stabilization temperature improved aromatization through nitrile loss (Figure 3iv). The lower content of carbon in the CNF_260 sample was reasonably due to the higher oxidation induced by the increment of the stabilization temperature as proven by the higher content of oxygen compared to CNF_230 (7.5 at.% and 6.1 at.% respectively obtained by the XPS analysis).

### 3.2. CO_2_ Adsorption Performance

**Figure 7:**
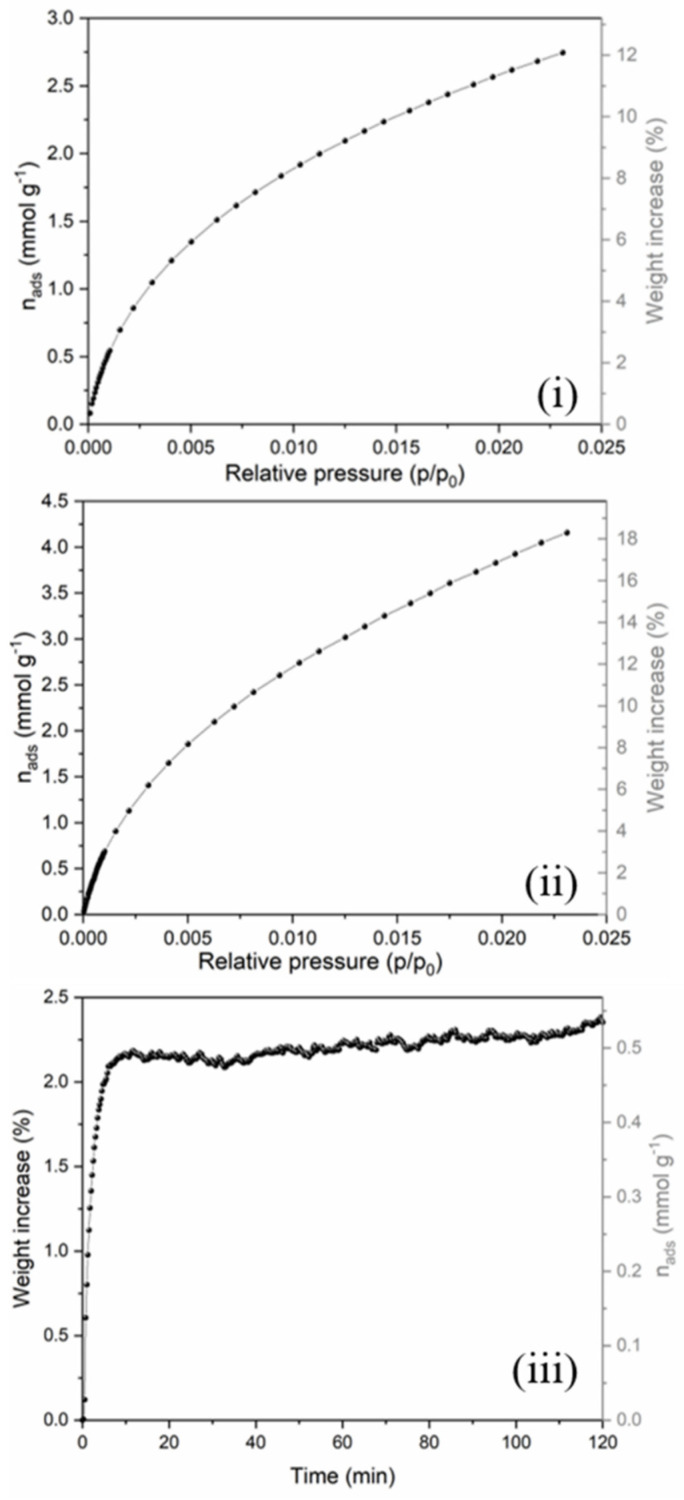
CO_2_ adsorption isotherms of (**i**) CNF_230 and (**ii**) CNF_260 at 20 °C in pure CO_2_ atmosphere and (**iii**) CO_2_/N_2_ selectivity test of CNF_230 mat at 30 °C in a simulated post-combustion flue gas mixture of 20 vol.% CO_2_ and 80 vol.% of N_2_.

The CO_2_ adsorption performance was analyzed by using a volumetric analyzer at 20 °C (see Figure 7i,ii). CNF_230 and CNF_260 samples showed a very similar adsorption behavior, but with better CO_2_ uptake for the latter, i.e., 4.16 mmol g^−1^ (18.3 wt.%) with respect to 2.75 mmol g^−1^ (12 wt.%) of CNF_230 sample. At a low pressure these two samples showed a very similar adsorption, while at a higher pressure the CO_2_ uptake of CNF_260 increased quickly. Some results on control samples are reported in the Supplementary Information file. An “as spun” (not stabilized) sample (AS) was not efficient in CO_2_ adsorption (0.3 mmol g^−1^ of adsorbed gas) due to inadequate/absent carbon fiber formation, no porous structure, and likely no residual N groups of the kind N6, N5 and NQ. The stabilized but not carbonized sample (STAB) showed a modest increment in CO_2_ adsorption compared with the AS sample. The histogram reported in SI (Figure S3) clearly shows the adsorption trend of the samples. TGA method was also applied on CNF_230 sample in order to assess the selectivity for CO_2_ adsorption in a CO_2_/N_2_ gas mixture, whose composition simulates a typical post-combustion flue gas [38]. Accordingly, the measurements were taken under 20% CO_2_ in N_2_ flow, and the result is shown in Figure 7iii. The weight of the CNF_230 sample increased rapidly as soon as it was exposed to the CO_2_ flux and after saturation, the CO_2_ uptake remained constant, which means that the molecules were physically adsorbed on the surface of the CNFs. The CO_2_ adsorbed amount reached 0.53 mmol g^−1^ (23 mg g^−1^), approximately 20% of the amount captured under a pure CO_2_ flow. This evidence is in good agreement with the tests performed under pure CO_2_, considering the corresponding CO_2_ partial pressure and the slight increase of the tested temperature (from 20 to 30 °C, for pure CO_2_ and CO_2_/N_2_ adsorption, respectively), which typically causes a decrease in the adsorption for physisorption processes [44]. Moreover, this result confirms the negligible N_2_ uptake in respect to CO_2_ under the tested conditions.

Some meaningful recent literature results are collected in Table 2. A variety of preparation methods have been explored in which the activation of the CNFs was also used. The adsorption performances achieved by our samples are among the best reported so far that can be beneficial from an environmental perspective and additionally advantageous from the manufacturing viewpoint, including resources and energy employed during the fabrication. Looking at these results, the overall content of N does not seem to be the main characteristic that drives the adsorption performances. Rather, the relative content of nitrogen-containing groups might clarify the superior CO_2_ adsorption of CNF_260, likely resulting from the combination of high amount of NQ with N6 groups, these latter quite comparable in both the samples (36% in CNF_230 vs. 32% in CNF_260).

The presence of pyrrolic moieties seemed to have less impact in this case compared to other literature results [32], but in agreement with other observations on PAN samples carbonized at different temperatures that showed a predominance of pyridinic and quaternary groups that facilitated CO_2_ adsorption [35].

Noteworthy, theoretical calculation demonstrated that NQ doped edge and basal planes positions contribute significantly to the adsorption properties, thus confirming the synergistic effect of the co-presence of NQ and N6 groups [42] that was beneficial for the CO_2_ uptake measured for CNF_260. The good amount of N6 and especially of NQ could be reasonably due to higher stabilization temperature of CNF_260.

According to the mechanism reported in Figure 3, during the cyclization process, the nitrile residues condensate into aromatic structures rich in pyridine moieties that can be rearranged in NQ groups as confirmed by our FT-IR results. Therefore, a more suitable precursor for the production of carbon rich in nitrogen basic sites was obtained, that interacted more efficiently with CO_2_ [42,48].

## 4. Conclusions

Among various sorbent materials, electrospun CNFs can be an excellent solution for carbon dioxide adsorption due to the easy, fast, and low-cost fabrication method. In this work, robust PAN nanofibers mats were produced at two different stabilization temperatures, namely 230 °C and 260 °C, while the carbonization step was set at 900 °C. The CNFs-based membranes were tested for CO_2_ adsorption in pure CO_2_ and in a N_2_/CO_2_ mixture for gas separation applications, and the favorable effect of a higher stabilization temperature was proven. The improvement of the adsorption efficiency is ascribed to the formation of a stabilized precursor rich in nitrogen containing condensed carbon structures that could be turned into N6 and NQ rich carbon materials. The massive presence of nitrogen basic sites is the key to the best performances showed by CNF_260 compared to CNF_230. The combination of a facile production technique such as the electrospinning coupled with the temperature tunable properties of carbonized PAN opens the way to further engineering of this material aiming to reach even higher performances. The interesting characteristics including the mechanical robustness of PAN-derived carbon fibers would make possible the industrial fabrication of large area, self-standing membranes which could reduce CO_2_ emissions from large point emission sources with minimal intervention on plants.

## Figures and Tables

**Table 1:** Characteristic features from XPS and Raman analysis of CNF_230 and CNF_260.

	Carbon (%)			Oxygen (%)		Nitrogen (%)			I_D_/I_G_
	C sp^2^ 284.8 eV	C-X (X=O, N) 287.8 eV	COOH 289.8 eV	C-OH 530.7 eV	COOH 532.8 eV	N6 398.2 eV	N5 399.5 eV		NQ 401.1 eV
CNF_230	58	38	4	14	86	36	22	43	1.34
CNF_260	44	43	13	11	89	32	14	53	1.04

**Table 2:** Adsorption performances at different conditions, preparation steps, and nitrogen content of literature samples compared to this work.

Preparation ^REF.^	Samples ^REF.^	N Content %	CO_2_ Capacity mmol·g^−1^	T_ads_ ^b^ °C	P_ads_ ^c^ bar	T_act_ ^d^ °C
Electrospinning Stabilization Carbonization	CNF_230 ^this work^	7.1 ^a^	0.53	35	1	150
			(CO_2_:N_2_ = 20:80)			
			2.75	20	1	
			(100% CO_2_)			
Electrospinning Stabilization Carbonization	CNF_260 ^this work^	6.6 ^a^		35	1	150
			(CO_2_:N_2_ = 20:80)			
			4.16	20	1	
			(100% CO_2_)			
Addition of pore forming agent (PVP ^d^) Cross-linking by HH ^e^ Electrospinning Stabilization Carbonization	PCF-H5 [32]	16.48 ^b^	0.73	25	1	110
			(CO_2_:N_2_ = 10:90)			
			2.29	25	1	200
			(100% CO_2_)			
Addition of pore forming agent (PVP ^d^) Electrospinning Carbonization	PCNF-2-1000 [45]	9.08 ^a^	3.11	25	1	105
			(100% CO_2_)			
Electrospinning Stabilization Carbonization CO_2_ activation	AFH2 [46]	9.0 ^a^	3.17	25	1	350
			(100% CO_2_)			
Electrospinning Stabilization Carbonization KOH activation	PAN-PK [30]	8.13 ^b^	4.4	25	1	150
			(100% CO_2_)			
Urea doping Electrospinning Carbonization CO_2_ activation	N-AnF(1:5) [43]	m.i. ^c^	2.98	m.i. ^c^	m.i. ^c^	m.i. ^c^
			(100% CO_2_)			
Melamine doping Electrospinning Stabilization CO_2_ activation	MACNF-7 [47]	m.i. ^c^	1.22	25	1	120
			(CO_2_:N_2_ = 15:85)			
	MACNF-10 [47]		3.15	0	1	m.i. ^c^
			(100% CO_2_)			

^a^ determined by XPS (at.%); ^b^ determined by elemental analysis (wt.%); ^c^ m.i. denotes missing information; ^d^ PVP: polyvinylpyrrolidone; ^e^ HH: hydrazine hydrate.

## Data Availability

The data presented in this study are available on request from the corresponding author.

## Supplementary Materials

The following are available online at https://www.mdpi.com/article/10.3390/polym13234197/s1; Figure S1: Survey spectra of (a) CNF_230 and (b) CNF_260; Figure S2: CO_2_ adsorption isotherms of the as-spun (namely, AS, i) and stabilized mats (namely, STAB, ii) in pure CO_2_ atmosphere; Figure S3: Histogram of the total amount of adsorbed CO_2_ (mmol g^−1^ and wt.%) for each sample.
