# Supplementary material for: Effect of Thermal Stabilization on PAN-Derived Electrospun Carbon Nanofibers for CO2 Capture
Source: Polymers (Basel). 2021 Nov 30;13(23):4197. doi: 10.3390/polym13234197 (PMC8659445; doi:10.3390/polym13234197)
Supplement: Supplementary file 1 [file polymers-13-04197-s001.zip › polymers-1453565-supplementary.pdf]

# Supplementary Information

## Effect of thermal stabilization on PAN-derived electrospun carbon nanofibers for $CO_2$ capture

E. Maruccia, S. Ferrari, M. Bartoli, L. Lucherini, G. Meligrana, C.F. Pirri, G. Saracco, C. Gerbaldi

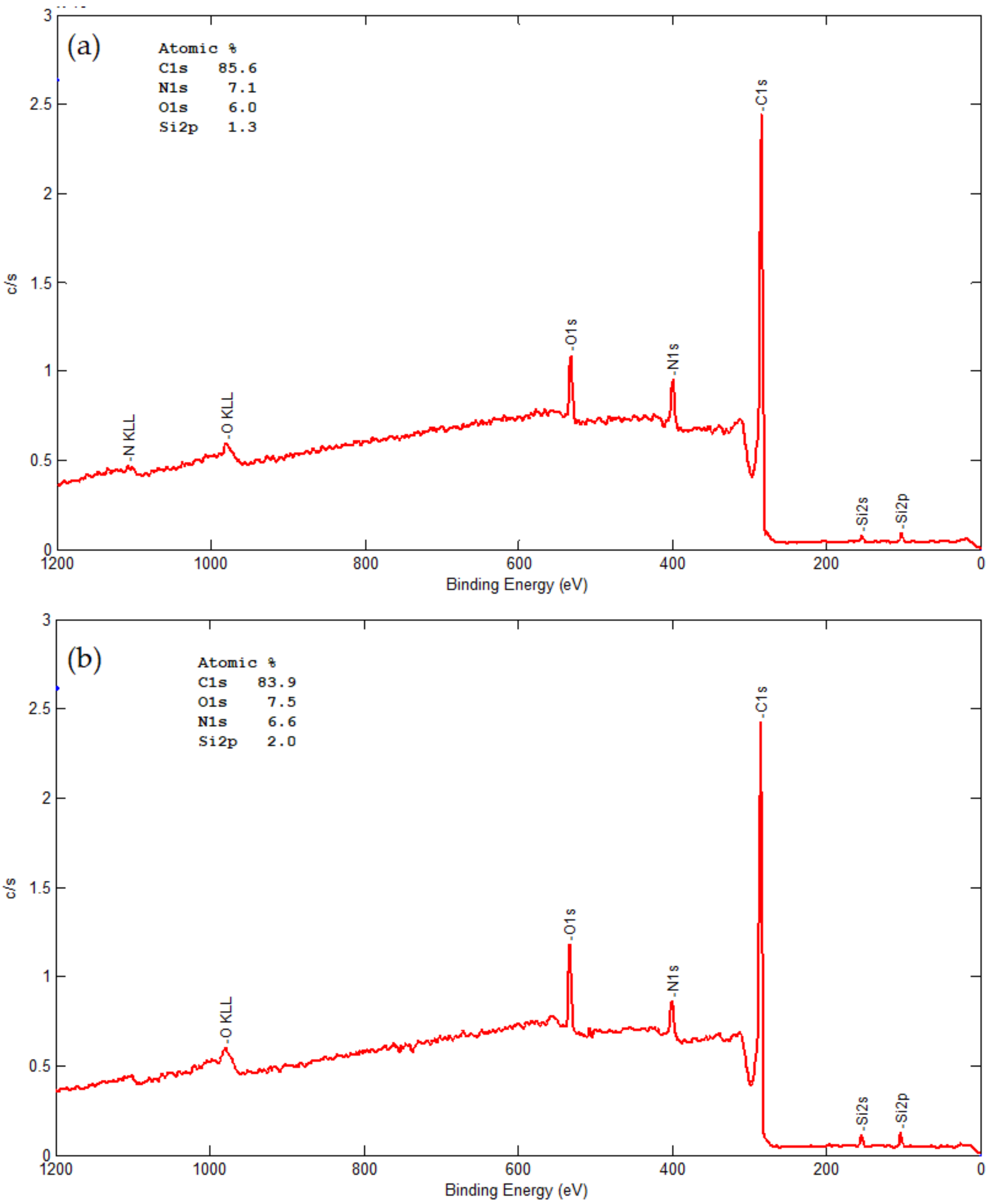


**Figure S1:** Survey spectra of a) CNF_230 and b) CNF_260.

**Control samples, experimental details.**

After the preparation by electrospinning (using the same conditions as for the main samples), the as spun (i.e., not thermally treated) and only stabilized (i.e. not carbonized) carbon mats were also considered for $CO_2$ capture characterization. The mentioned samples have been named as:

- **AS**, no thermal treatments (sample "as spun");
- **STAB**, stabilized in air @ 100, 200 °C for 30 min and @ 260 °C for 120 min, without carbonization.

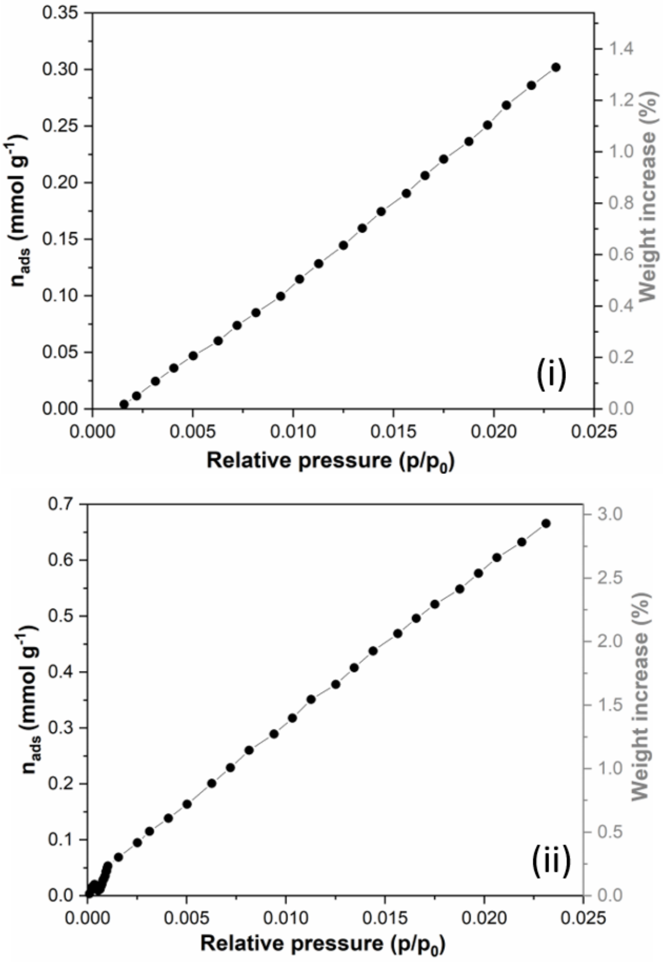


**Figure S2:** $CO_2$ adsorption isotherms of the as-spun (namely, AS, i) and stabilized mats (namely, STAB, ii) in pure $CO_2$ atmosphere.

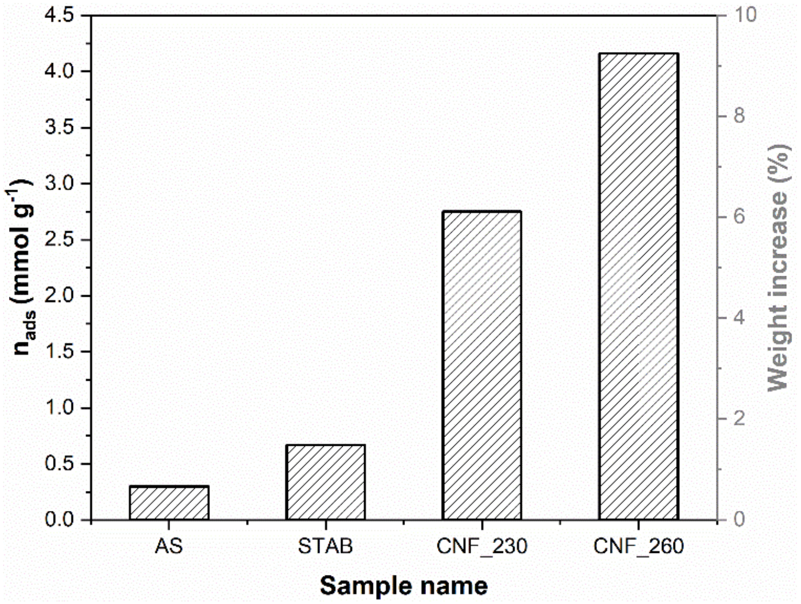


**Figure S3**: Histogram of the total amount of adsorbed $CO_2$ (mmol $g^{-1}$ and wt%) for each sample.
